# Detection Rates and Trends of Asymptomatic Unruptured Intracranial Aneurysms From 2005 to 2019

**DOI:** 10.1227/neu.0000000000002664

**Published:** 2023-09-11

**Authors:** Dan Laukka, Juri Kivelev, Melissa Rahi, Tero Vahlberg, Jooa Paturi, Jaakko Rinne, Jussi Hirvonen

**Affiliations:** * Department of Neurosurgery, Neurocenter, Turku University Hospital, Turku, Finland;; ‡ Clinical Neurosciences, University of Turku, Turku, Finland;; § Department of Clinical Medicine, Biostatistics, University of Turku, Turku, Finland;; ‖ Department of Radiology, Turku University Hospital and University of Turku, Turku, Finland;; ¶ Department of Radiology, University of Tampere, Tampere, Finland;; # Department of Biostatistics, University of Turku and Turku University Hospital, Turku, Finland

**Keywords:** Detection rate, Trends, Unruptured intracranial aneurysm, Computed tomography angiography, Magnetic resonance angiography

## Abstract

**BACKGROUND AND OBJECTIVES:**

The trend in detection rates of asymptomatic unruptured intracranial aneurysms (UIAs) on brain computed tomography angiography/magnetic resonance angiography (CTA/MRA) is not well established. Our objective was to evaluate time trends in asymptomatic UIA detection rates on brain CTA/MRA between 2005 and 2019.

**METHODS:**

We conducted a retrospective study of all brain computed tomography/magnetic resonance scans (n = 288 336 scans in 130 621 patients) performed between January 2005 and December 2019 at a tertiary referral hospital. Patients who underwent brain CTA/MRA examinations were included (n = 81 261 scans in 48 037 patients). The annual detection rate of new UIA cases was calculated based on the first brain CTA/MRA imaging. Detection rates were compared between three periods and across different age groups.

**RESULTS:**

The number of first CTA/MRA examinations increased significantly from 2005 to 2009 (n = 12 190 patients) to 2010–2014 (n = 14 969 patients) and 2015–2019 (n = 20 878 patients) (*P* < .001). The UIA detection rate also increased significantly from 1.7% in 2005–2009 to 2.5% in 2010–2014 and 3.4% in 2015–2019 (*P* < .001). The UIA detection rate increased significantly from 2010–2014 to 2015–2019 (relative risk [RR], 1.33; 95% CI, 1.17-1.51), particularly in patients aged 60–69 years (RR, 1.29; 95% CI, 1.01-1.63), 70–79 years (RR, 1.71; 95% CI, 1.30-2.25), and >79 years (RR, 2.33; 95% CI, 1.56-3.47). Furthermore, the detection rate of <5-mm UIAs increased from 2010–2014 to 2015–2019 (RR, 1.51; 95% CI, 1.28-1.77).

**CONCLUSION:**

The detection rate of asymptomatic UIAs, particularly in elderly patients, has increased significantly over the past 15 years, coinciding with the increased use of CTA/MRA imaging. Furthermore, the size of the identified UIAs has decreased. These findings raise concerns about the management strategies for UIAs, indicating the need for further research.

## Graphical abstract

**Figure:**
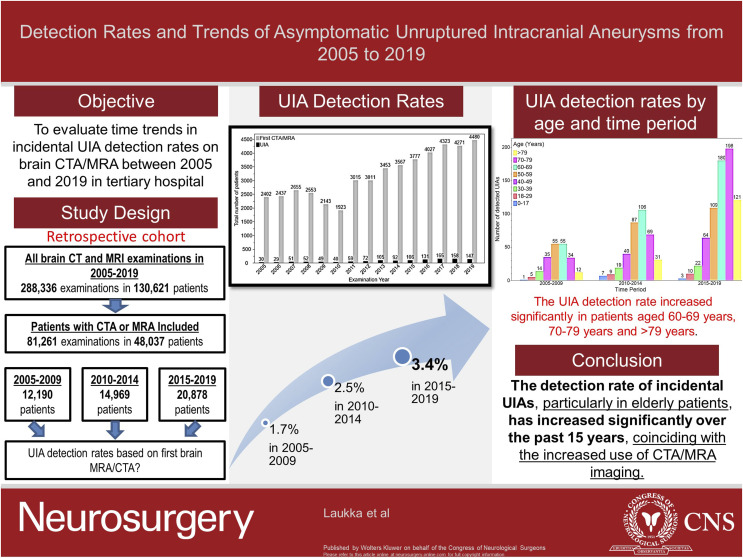

**ABBREVIATIONS:**
MRA: magnetic resonance angiography
RR: relative risk.

The prevalence of unruptured saccular intracranial aneurysms (UIA) in the general population is approximately 3%,^1^ and aneurysmal subarachnoid hemorrhage has a worldwide incidence of 6 cases per 100 000 people.^2^ Aneurysmal subarachnoid hemorrhage mainly affects the working-age population and is associated with significant disability and mortality.^2^ However, the treatment of incidentally discovered UIAs remains controversial^3^ because most of the UIAs, especially small ones, do not rupture during an individual's lifetime.^4^

With the increase in neuroimaging, particularly in the elderly,^5^ the number of incidentally discovered UIAs is likely to rise. Furthermore, the aging population and increased life expectancy^6^ present a significant clinical challenge when a UIA is incidentally detected because the incidence of aneurysmal subarachnoid hemorrhage is increasing in older adults.^7,8^

While previous studies have examined trends in subarachnoid hemorrhage^7,8^ and the prevalence of asymptomatic UIAs,^9-12^ there has been a lack of new studies investigating whether the prevalence of asymptomatic UIAs has changed over time in the current era and in different age groups in relation to the increased use of computed tomography angiography (CTA) and MRI angiography (MRA).^13^

This retrospective cohort study aimed to evaluate the detection rate of asymptomatic UIAs by reviewing all brain CTA and MRA examinations conducted at our tertiary hospital between 2005 and 2019.

## METHODS

The study was approved by the local institutional review board. Patient consent was not required because of the study's retrospective design.

We manually reviewed radiological reports of all brain computed tomography (CT) and magnetic resonance imaging (MRI) scans conducted at our tertiary hospital between 2005 and 2019. Identification of the study population and the flow chart are presented in **Supplemental Digital Content 1, Methods** (http://links.lww.com/NEU/D919).

We reviewed a total of 130 621 patients with 182 436 brain CT and 105 900 brain MRI examinations to determine whether angiography had been performed. We excluded patients who had not undergone CTA or MRA imaging between 2005 and 2019, as well as those with arteriovenous malformation/fistula (n = 276), exclusively fusiform aneurysms (n = 53), mycotic aneurysms (n = 2), intracranial pseudoaneurysms (n = 7), nonaneurysmal subarachnoid hemorrhage (SAH) (n = 124), aneurysmal SAH (n = 1479), symptomatic UIAs (n = 27), previously diagnosed UIAs (n = 138), and UIAs diagnosed outside (n = 426) our tertiary hospital.

All radiological reports of brain CTA and MRA from the included patients were reviewed manually to identify detected UIA. UIAs were defined as saccular aneurysms with a size of ≥1 mm. In this cohort, there were no <1-mm UIAs reported in the CTA or MRA scans. We analyzed the maximum UIA size, the number of UIAs, and the location of UIAs from the radiological reports. The patient's sex and age for each calendar year of brain CTA/MRA imaging were analyzed. According to Bouthillier classification,^14^ aneurysms located in and distal to the clinoid segment (C5) were defined as intradural; aneurysms located in the intracavernous segment (C4) were classified as extradural. Aneurysms located proximal to the C4 segment were excluded from analysis. UIA size and location classifications are presented in **Supplemental Digital Content 1, Methods** (http://links.lww.com/NEU/D919).

We calculated the number of new patients for each calendar year based on first brain CTA/MRA examination or the year when the UIA was first detected from the brain CTA/MRA examination. The first CTA/MRA was the calendar year when the patient had their first brain CTA or MRA or when the UIA was first diagnosed. Multiple brain MRA/CTA examinations were considered as one case.

We also separately calculated the cumulative number of brain CTA and MRA examinations and the number of patients for each calendar year. In these calculations, we included every patient for each year, regardless of whether the patient had been included in other years as well. If a patient had multiple brain MRA or CTA scans in the same calendar year, that patient was considered as one case in that calendar year when calculating the cumulative case volume.

### Statistical Analysis

All analyses were conducted using SAS System for Windows (version 9.4, SAS Institute Inc.).

The study population was subdivided into different age groups (<18, 18-29, 30-39, 40-49, 50-59, 60-69, 70-79, and >79 years) and quartiles based on the year when the brain CTA and MRA were performed (2005-2009, 2010-2014, and 2015-2019). The sizes of UIAs were classified into 3 categories: ≥1  mm, ≥2  mm, and ≥3  mm. In addition, when computing the distribution and detection rate of small aneurysms across different periods, a UIA size of less than 5 mm was categorized as <5 mm and a UIA size of less than 7 mm was classified as <7 mm.

We calculated the detection rate of UIAs for each calendar year based on the year of the patient's first brain CTA/MRA examination or the year of the UIA's first diagnosis. The detection rates were computed using different definitions for UIA size (≥1  mm, ≥2  mm, and ≥3  mm) and location (intradural and extradural or only intradural). Extradural locations were excluded from the analysis when calculating intradural aneurysms separately.

Categorical data including age groups, the year of brain CTA/MRA, the presence of UIA, sex, and the location of UIA were compared using the χ^2^-test. The normality of continuous data such as age and aneurysm size was assessed visually using histograms. The Kruskal–Wallis test was used to compare the size of the UIAs between different periods and age groups. Mean ages between periods and age groups were compared using one-way analysis of variance.

The UIA detection rate was analyzed using generalized linear models with a binomial distribution and a log link. The UIA detection rates between different periods were adjusted with age and sex. Nonadjusted UIA detection rates and the number of CTA/MRA examinations were analyzed separately for individuals in the various age groups between different periods. When analyzing the difference in UIA detection rate between females and males, the calculations were adjusted with age groups and periods and separately for an interaction with age groups.

*P*-values of <.05 were considered statistically significant. Missing data were not included in the analysis.

## RESULTS

A total of 48 037 patients who underwent 49 678 brain MRA and 31 589 brain CTA examinations were included in this study. The mean age (at first CTA/MRA scan) of the patients was 55.1 years (SD 21.2), and 54.2% of the patients were female (n = 26 026). A total of 1544 UIAs were found in 1286 patients (2.7%).

### UIA Detection Rate in Different Periods

**FIGURE 1.**
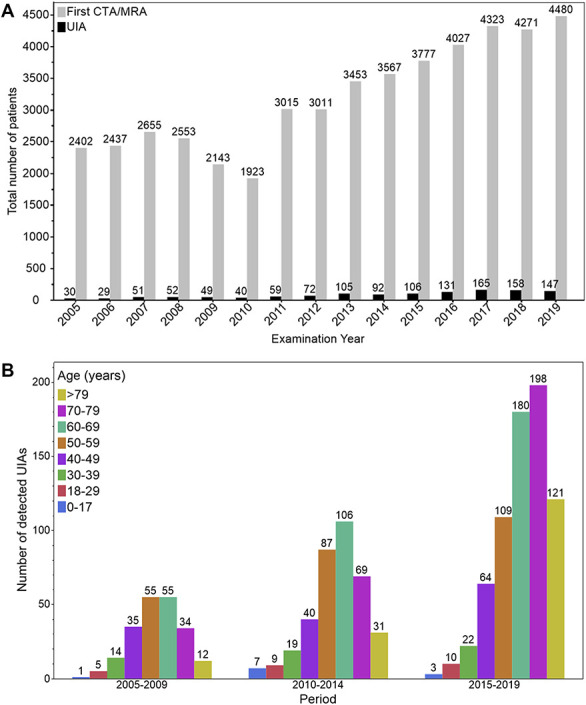
Detection rates of UIAs. **A**, Number of patients undergoing first CTA/MRA imaging each year from 2005 to 2019. The gray bars correspond to the number of new UIA cases diagnosed each year, whereas the black bars represent the number of patients who had their first CTA/MRA examination in that year. The numbers above the bars indicate the exact values of each category. **B**, Number of new patients diagnosed with UIA by age group and period. The data are presented as a bar graph with the age group on the x-axis and the number of new cases on the y-axis. The period is divided into three groups: 2005–2009, 2010–2014, and 2015–2019. The bars are color-coded by age group. CTA, computed tomography angiography; MRA, magnetic resonance angiography; UIA, unruptured intracranial aneurysm.

Table 1 presents the detection rates of UIAs in different periods, and Figure 1A shows the rates for each calendar year. The mean age at the diagnosis of UIA increased from 58.2 (SD 14.0) years in 2005–2009 and 59.9 (SD 15.9) years in 2010–2014 to 66.1 (SD 14.7) years in 2015–2019 (*P* < .001). Women had a higher overall detection rate of UIAs than men, with rates of 3.2% and 2.1%, respectively (<.001) (Table 1).

**TABLE 1.** Patient Characteristics and Detection Rates of Saccular UIAs From First CT Angiography or MR Angiography in Different Periods and Cumulative Number of Imaging Performed: RRs (95% CI) Adjusted with Age and Sex in 2015–2019 Compared With 2010–2014

Characteristics	Total	Period, y			RR 2015–2019 vs 2010–2014	*P*-value
		2005–2009	2010–2014	2015–2019		
First CTA or MRA—No. of patients	48 037	12 190	14 969	20 878	—	<.001
CTA—No. of patients (%)	17 278 (36.0)	4620 (37.9)	5088 (34.0)	7570 (36.3)	1.09 (1.04-1.15)	<.001
MRA—No. of patients (%)	26 035 (54.2)	6817 (55.9)	8585 (57.4)	10 633 (50.9)	ref	
Both—No. of patients (%)	4724 (9.8)	753 (6.2)	1296 (8.6)	2675 (12.8)	—	
Mean age—years (SD						
Total	55.1 (21.2)	52.5 (21.0)	54.5 (21.3)	57.1 (21.1)	—	<.001
Without UIA	63.0 (15.3)	52.4 (21.1)	54.3 (21.4)	56.8 (21.2)	—	<.001
With UIA	54.9 (21.3)	58.2 (14.0)	59.9 (15.9)	66.1 (14.7)	—	<.001
Sex—No. (%)						
Female, total	26 026 (54.2)	6651 (54.6)	8154 (54.5)	11 221 (53.7)	0.97 (0.93-1.01)	.11
Male, total	22 011 (45.8)	5539 (45.4)	6815 (45.5)	9657 (46.3)	ref	
UIA detection by imaging type						
CTA—No. (%)	622/1286 (48.4)	88/211 (41.7)	146/368 (39.7)	388/707 (54.9)	1.51 (1.15-1.98)	.003
MRA—No. (%)	664/1286 (51.6)	123/211 (58.3)	222/368 (60.3)	319/707 (45.1)	ref	
Detection rate of intradural/extradural UIAs—total No. of patients included (%)						
≥1 mm	1286/48 037 (2.7)	211/12 190 (1.7)	368/14 969 (2.5)	707/20 878 (3.4)	1.33 (1.17-1.51)	<.001
≥2 mm	1262/48 013 (2.6)	210/12 189 (1.7)	363/14 964 (2.4)	689/20 860 (3.3)	1.31 (1.15-1.49)	<.001
≥3 mm	943/47 694 (2.0)	166/12 145 (1.4)	274/14 875 (1.8)	503/20 674 (2.4)	1.26 (1.09-1.46)	<.001
<5 mm	811/48 037 (1.7)	108/12 190 (0.9)	223/14 601 (1.5)	480/20 878 (2.3)	1.51 (1.28-1.77)	<.001
<7 mm	1053/48 037 (2.2)	154/12 190 (1.3)	303/14 969 (2.0)	596/20 878 (2.9)	1.37 (1.19-1.58)	<.001
Intradural UIAs—No. of patients (%)						
≥2 mm	1146/47 897 (2.4)	189/12 168 (1.6)	326/14 927 (2.2)	631/20 802 (3.0)	1.33 (1.16-1.52)	<.001
≥3 mm	886/47 637 (1.9)	155/12 134 (1.3)	256/14 857 (1.7)	475/20 646 (2.3)	1.27 (1.09-1.48)	.002
Extradural UIAs only—No. of patients (%)	119/48 037 (0.3)	21/12 190 (0.2)	37/14 969 (0.3)	61/20 878 (0.3)	1.00 (1.00-1.00)	1.0
UIA detection rate by sex						
UIA in female	833 (3.2)^a^	127 (1.9)	244 (3.0)	462 (4.1)	0.93 (0.71-1.22)	.59
UIA in male	453 (2.1)^a^	84 (1.5)	124 (1.8)	245 (2.5)	ref	
Total No. of imaging						
Total—No.	81 267	15 972	23 737	41 588		<.001
CTA—No. (% of total)	31 589	6460 (40.4)	8648 (36.4)	16 481 (39.7)	1.03 (0.99-1.06)	.17
MRA—No. (% of total)	49 678	9512 (59.6)	15 089 (63.6)	25 077 (60.3)	ref	

CTA, computed tomography angiography; MRA, magnetic resonance angiography; RR, relative risk; UIA, unruptured intracranial aneurysm.

a *P*-value <.001 between female and male.

The detection rate of any UIAs ≥1 mm increased in three periods: from 1.7% in 2005–2009 to 2.5% in 2010–2014 and 3.4% in 2015–2019. In 2015–2019, compared with 2010–2014, the detection rate of any ≥1-mm UIAs increased significantly (age-/sex-adjusted relative risk [RR], 1.33; 95% CI, 1.17-1.51). A similar increase was seen in ≥2-mm and ≥3-mm intradural and/or extradural UIAs and intradural UIAs (Table 1).

The detection rate of small <5-mm UIAs increased significantly in 2015–2019 when compared with 2010–2014 (age-/sex-adjusted RR, 1.51; 95% CI, 1.28-1.77) (Table 1).

The UIA detection rate increased especially in elderly (Figure 1B). When comparing the detection rates of asymptomatic UIAs for two periods, 2015–2019 and 2010–2014, there were evident increases in the following age groups: 60–69 years (sex-adjusted RR, 1.29; 95% CI, 1.01-1.63), 70–79 years (sex-adjusted RR, 1.71; 95% CI, 1.30-2.25), and >79 years (sex-adjusted RR, 2.33; 95% CI, 1.56-3.47) (Table 2).

**TABLE 2.** RR (95% CI) Adjusted With Sex for the Detection Rates of Saccular UIAs (UIA) and the Number of Patients Having First CTA/MRA for Each Age Group in the Different Periods

Age group, years	2015–2019 vs 2010–2014	2015–2019 vs 2005–2009	2010–2014 vs 2005–2009
Detection rate of all UIAs			
0–17	0.38 (0.10-1.49)	2.42 (0.25-23.30)	6.31 (0.78-51.17)
18–29	0.87 (0.36-2.14)	1.35 (0.46-3.92)	1.54 (0.52-4.60)
30–39	0.90 (0.49-1.66)	1.08 (0.56-2.11)	1.20 (0.60-2.37)
40–49	1.30 (0.88-1.93)	1.38 (0.92-2.09)	1.06 (0.68-1.67)
50–59	0.93 (0.71-1.23)	**1.58 (1.14-2.19)**	**1.69 (1.21-2.37)**
60–69	**1.29 (1.01-1.63)**	**1.66 (1.23-2.23)**	1.29 (0.93-1.78)
70–79	**1.71 (1.30-2.25)**	**2.60 (1.81-3.75)**	**1.52 (1.01-2.30)**
>79	**2.33 (1.56-3.47)**	**3.63 (2.02-6.55)**	1.56 (0.80-3.04)
First CTA/MRA			
0–17	**0.79 (0.72-0.86)**	**0.71 (0.65-0.77)**	**0.90 (0.82-0.98)**
18–29	**0.91 (0.84-0.98)**	**0.86 (0.79-0.93)**	0.95 (0.87-1.03)
30–39	**0.91 (0.85-0.98)**	**0.84 (0.77-0.90)**	**0.91 (0.84-0.99)**
40–49	**0.85 (0.80-0.91)**	**0.76 (0.71-0.81)**	**0.89 (0.83-0.96)**
50–59	**0.94 (0.89-0.99)**	**0.71 (0.67-0.75)**	**0.75 (0.71-0.80)**
60–69	**0.92 (0.87-0.97)**	**1.18 (1.11-1.25)**	**1.28 (1.20-1.36)**
70–79	**1.28 (1.21-1.35)**	**1.41 (1.33-1.49)**	**1.10 (1.03-1.18)**
>79	**1.32 (1.23-1.42)**	**1.70 (1.57-1.84)**	**1.29 (1.19-1.41)**

CTA, computed tomography angiography; MRA, magnetic resonance angiography; RR, relative risk; UIA, unruptured intracranial aneurysm.

Numbers in bold indicate statistically significant results (*P* < .05).

### UIA Characteristics in Different Periods and Age Groups

The mean maximum diameter of UIAs at the time of first diagnosis decreased steadily over time, from 5.7 mm (SD 4.4) in 2005–2009 to 4.8 (SD 3.5) in 2010–2014 and 4.2 (SD 2.7) in 2015–2019 (*P* < .001) (Table 3).

**TABLE 3.** Characteristics of Saccular UIA From First CTA or MRA in Different Periods: RR (95% CI) in 2015–2019 Compared With That in 2010–2014

Characteristics	Total	Period, y			RR (95% CI) 2015–2019 vs 2010–2014	*P*-value
		2005–2009	2010–2014	2015–2019		
Largest UIA—No.	1286	211	368	707	—	**<.001**
Size of the largest aneurysm—mean, mm (SD)	4.6 (3.3)	5.7 (4.4)	4.8 (3.5)	4.2 (2.7)	—	**<.001**
Distribution of the largest aneurysm—No. (%)						
1–2 mm	342 (26.6)	45 (21.3)	94 (25.5)	203 (28.7)	1.29 (0.97-1.74)	.08^b^
3–4 mm	469 (36.5)	63 (29.9)	129 (35.1)	277 (39.2)	1.10 (0.85-1.43)	.47^b^
5–6 mm	242 (18.8)	46 (21.8)	80 (21.7)	116 (16.4)	**0.68 (0.49-0.94)**	**.02** ^b^
7–9 mm	154 (12.0)	29 (13.7)	36 (9.8)	89 (12.6)	1.17 (0.79-1.77)	.42^b^
≥10 mm	79 (6.1)	28 (13.3)	29 (7.9)	22 (3.1)	**0.32 (0.18-0.58)**	**<.001** ^b^
Proportion of <5 mm aneurysms—No. (%)	811 (63.1)	108 (51.2)	223 (60.6)	480 (67.9)	**1.55 (1.18-2.03)**	**.002** ^b^
Proportion of <7 mm aneurysms—No. (%)	233 (18.1)	57 (27.0)	65 (17.7)	111 (15.7)	1.30 (0.92-1.83)	.13^b^
Multiple aneurysms—No. of patients (%)	239/1286 (18.5)	54/211 (16.9)	71/368 (19.5)	109/707 (15.6)	0.92 (0.66-1.27)	.61
Location of the largest aneurysms—No. (%)						
ICA	323/1286 (25.1)	39/211 (18.5)	86/368 (23.4)	198/707 (28.0)	**1.34 (1.00-1.81)**	**.049**
MCA	465/1286 (36.2)	76/211 (36.0)	134/368 (36.4)	255/707 (36.1)	1.0 (1.0-1.0)	1.0
ACA^a^	261/1286 (20.3)	53/211 (25.1)	71/368 (19.3)	137/707 (19.4)	1.0 (1.0-1.0)	1.0
Posterior circulation	118/1286 (9.2)	22/211 (10.4)	40/368 (10.9)	56/707 (7.9)	0.91 (0.59-1.39)	.66
Extradural	119/1286 (9.3)	21/211 (10.0)	134/368 (10.1)	61/707 (8.6)	1.0 (1.0-1.0)	1.0
Location of all aneurysms—No. (%)						
Total—No.	1544	249	449	846		
ICA	397/1544 (25.7)	47/249 (18.9)	114/449 (25.4)	236/846 (27.9)	1.14 (0.88-1.48)	.33
MCA	540/1544 (35.0)	88/249 (35.3)	158/449 (35.2)	294/846 (34.8)	1.0 (1.0-1.0)	1.0
ACA^a^	306/1544 (19.8)	61/249 (24.5)	77/449 (17.1)	168/846 (19.9)	1.1 (0.82-1.48)	.53
Posterior circulation	144/1544 (9.3)	26/249 (10.4)	50/449 (11.1)	68/846 (8.0)	0.87 (0.60-1.28)	.49
Extradural	157/1544 (10.2)	27/249 (10.8)	50/449 (11.1)	80/846 (9.5)	1.0 (1.0-1.0)	1.0

ICA, intradural internal carotid artery; MCA, medial cerebral artery; RR, relative risk; UIA, unruptured intracranial aneurysm.

a ACA = anterior circulation including anterior cerebral arteries and pericallosal arteries.

b *P*-values and relative rates adjusted with age and sex.

Numbers in bold indicate statistically significant results (*P* < .05).

Comparing 2015–2019 with 2010–2014, the proportion of UIAs sized 5–6 mm and ≥10 mm significantly decreased (age/sex-adjusted RR 0.68; CI 95%, 0.49-0.94 and 0.32; 95% CI, 0.18-0.58, respectively) (Table 3).

In 2015–2019, compared with 2010–2014, the proportion of UIAs sized <5 mm significantly increased relative to those sized ≥5 mm (age-/sex-adjusted RR, 1.55; 95% CI, 1.18-2.03). The UIA detection rate significantly increased with age (*P* < .001), with the lowest detection rate in the age group of 0–17 years (0.4%) and the highest detection rate in the age group of 60–69 years (3.5%) (**Supplemental Digital Content 2, Table 1**, http://links.lww.com/NEU/D920).

Females had a higher UIA detection rate in every age group from 0–17 years to >79 years (RR adjusted with the period and age group, 1.66; 95% CI, 1.48-1.89) (**Supplemental Digital Content 2, Table 1**, http://links.lww.com/NEU/D920).

### First MRA/CTA Examinations in Different Periods

**FIGURE 2.**
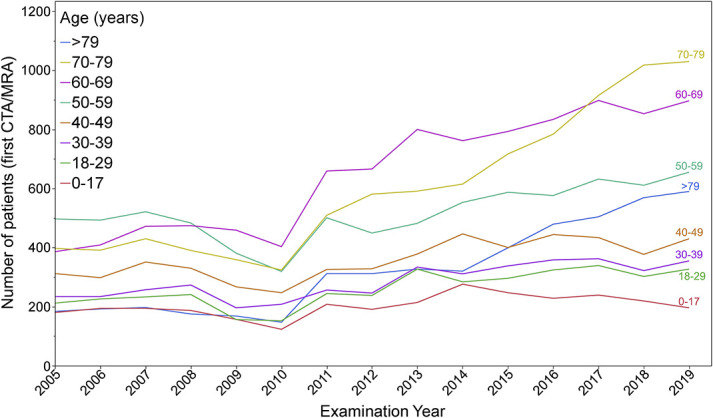
The number of new patients undergoing their first CTA/MRA imaging by age group and year from 2005 to 2019. This line graph shows the number of new patients undergoing their first CTA/MRA imaging, categorized by age group and year from 2005 to 2019. The lines are differentiated by color. CTA, computed tomography angiography; MRA, magnetic resonance angiography.

The total numbers of patients with the first CTA/MRA scans and for each calendar year are presented in Figure 1A and separately for each age group in Figure 2.

The number of first CTA/MRA examinations increased significantly over time, with a total of 12 190 patients in 2005–2009, 14 969 patients in 2010–2014, and 20 878 patients in 2015–2019 (*P* < .001). Furthermore, the mean age at the time of the first CTA/MRA examination increased significantly from 52.5 years (SD 21.0) in 2005–2009 and 54.5 years (SD 21.3) in 2010–2014 to 57.1 years (SD 21.1) in 2015–2019 (*P* < .001) (Table 1).

There was a significant increase in the imaging rate of the first CTA/MRA in the age groups of 70–79 years (sex-adjusted RR, 1.28; 95% CI, 1.21-1.35) and >79 years (sex-adjusted RR, 1.32; 95% CI, 1.23-1.42) when compared with the other age groups (Table 2).

### Cumulative Number of MRA and CTA Examinations in Different Periods

**FIGURE 3.**
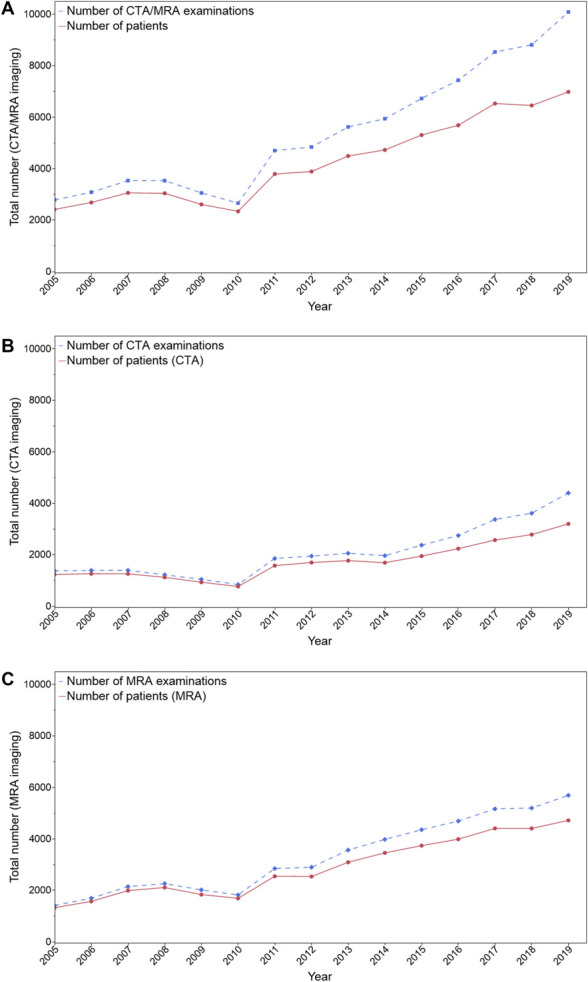
Cumulative number of patients and CTA/MRA examinations for each year from 2005 to 2019. **A**, Total number of CTA/MRA examinations and patients for each year. **B**, Total number of CTA examinations and patients for each year. **C**, Total number of MRA examinations and patients for each year. CTA, computed tomography angiography; MRA, magnetic resonance angiography.

The cumulative number of patients and the number of CTA and MRA examinations for each year in the period of 2005–2019 are presented in Figure 3A-3C, and separately for each age group in **Supplemental Digital Content 3, Figure 1** (http://links.lww.com/NEU/D921). The total number of CTA/MRA examinations increased significantly from 15 972 in 2005–2009 and 23 737 in 2010–2014 to 41 558 in 2015–2019 (*P* < .001).

Further analysis revealed that in the period of 2015–2019, compared with 2010–2014, the imaging rates for both MRA and CTA increased in two age groups in relation to other age groups: those older than 79 years (sex-adjusted RR for MRA, 1.42; 95% CI, 1.27-1.58 and sex-adjusted RR for CTA, 1.19; 95% CI, 1.11-1.27) and those aged 70–79 years (sex-adjusted RR for MRA, 1.43; 95% CI, 1.35-1.52 and sex-adjusted RR for CTA, 1.19; 95% CI, 1.12-1.26) as shown in **Supplemental Digital Content 4, Table 2** (http://links.lww.com/NEU/D922).

## DISCUSSION

From 2005 to 2019, the total number and detection rate of UIAs have increased over time in parallel with the greater number of CTA/MRA scans being conducted. The detection rate of UIAs and the number of brain MRA and CTA imaging have increased significantly in the elderly. The overall detection rate of all UIAs was 2.7%; when excluding extradural UIAs and <2-mm aneurysms, the overall detection rate of UIAs was 2.4%. The mean size of the detected UIAs has decreased over time. The detection rate of UIAs smaller than 5 mm has increased in the period of 2015–2019 when compared with earlier periods.

Our study found a similar overall detection rate of asymptomatic UIAs as in most previously published reports although we included brain CTA examinations in addition to brain MRA examinations. Earlier studies with a similar methodology as ours have reported prevalences of asymptomatic UIAs in brain MRA ranging from 1.8% to 3.5%.^9-12^ Our results are also similar to population-based studies by Vernooij et al (2007),^15^ Müller et al (2013),^16^ and Bos et al (2016),^17^ which reported prevalences of asymptomatic UIAs in the general population of 1.8%, 1.9%, and 2.3%, respectively. It is worth noting that in the studies by Vernooij^15^ and Bos,^17^ intracranial aneurysm diagnosis was based on native MRI studies without angiography sequences. In a recent study by Johnsen et al^18^ (2022), the prevalence of asymptomatic UIAs from brain MRA for different sizes of aneurysms and for extradural aneurysms was evaluated. The prevalence for ≥1-mm intradural and extradural aneurysms was 8.3%, and that for ≥2-mm intradural aneurysms was 6.6%, whereas for ≥3-mm intradural aneurysms, it was lower at 3.8%. In another population-based study, the prevalence of asymptomatic UIAs was estimated to be 7%.^19^ Differences in the included population and imaging techniques and the development of imaging techniques, may account for the differences in the reported prevalence of asymptomatic UIAs.

The most striking finding in our study was that brain CTA/MRA imaging and detection rate of asymptomatic UIAs have increased over time, especially in the elderly population. Specifically, from 2015 to 2019, the detection rate of UIAs increased notably among subjects aged over 70 years when compared with earlier periods. However, the detection rate of asymptomatic UIAs did not change significantly in patients aged 0–49 years between 2005 and 2019. Similar to earlier studies,^9,18,20^ we observed an age-related increase in the detection rate of asymptomatic UIAs, with the highest detection rate observed in individuals older than 50 years (3.2%-3.5%), with a peak in the age group of 60–69 years.

The incidence of aneurysmal SAH is increasing in the elderly,^7,8^ and higher age might increase the risk of UIA rupture even after the age of 80 years.^21^ However, the management of asymptomatic UIAs in the elderly population is controversial, because surgical procedures may pose a higher risk of complications in these individuals,^22^ although careful patient selection may decrease the risk of complications.^23^ Nevertheless, it seems that the number of detected UIAs in the elderly population is increasing, and the trend is likely to continue with the aging of the population and increased life expectancy.

As in previous studies,^4,19,24^ we also observed that most of the detected UIAs are small. We found that the mean size of detected UIAs has decreased over time and that the detection rate and the proportion of UIAs smaller than 5 mm have increased. Because aneurysms of size less than 5–7 mm are generally recommended to be managed conservatively, more aneurysms may require imaging surveillance,^3^ which could potentially result in an increased burden on healthcare budgets.

Based on our study, the number of discovered asymptomatic UIAs is increasing, particularly in the elderly population. It is crucial to develop more precise methods for assessing the risk of UIA rupture, which would aid in better resource allocation. In addition, it would be advantageous to have conservative treatment options for UIAs, such as medication,^25^ and new imaging modalities for evaluating the risk of UIA rupture.^26^

### Limitations

This study represents the largest hospital-based cohort study to date, evaluating the detection rate of UIAs. While the study has some limitations, such as its retrospective nature, which may introduce a selection bias, one of its strengths is that it analyzed every CTA and MRA scan performed at the hospital, reflecting actual clinical practice. However, because most UIAs are detected incidentally, the study population does not represent the general healthy population, but rather those with underlying diseases necessitating imaging. One of the strengths was a wide representation of different age groups, including patients younger than 18 years. Although not every CTA/MRA image was examined separately, the number of missed UIAs is likely small and unlikely to significantly affect the results. False-positive findings, especially with small UIAs, may also occur, but these typically do not prompt further investigation. Overall, the study's strengths and limitations provide valuable insights into the detection rate of UIAs in clinical practice.

## CONCLUSION

In the past 15 years, there has been a significant increase in the number and detection rate of asymptomatic UIAs in parallel with the increased use of brain CTA and MRA imaging. This increase is particularly notable in elderly populations, who are increasingly undergoing brain CTA and MRA imaging. As a result, a higher number of UIAs are being detected in the older population. In addition, there has been a rise in the detection of UIAs smaller than 5 mm. Our findings underscore the importance of developing standardized management guidelines for detected UIAs and further research to better understand their natural history.

## Supplementary Material

## SUPPLEMENTAL DIGITAL CONTENT

**Supplemental Digital Content 1. Supplemental Methods.** Additional details about the identification of the study population and aneurysm characteristics (http://links.lww.com/NEU/D919). **Supplemental Digital Content 2. Supplemental Table 1.** Patient characteristics, detection rate, and characteristics of saccular unruptured intracranial aneurysms (UIAs) from first CT angiography (CTA) or MR angiography (MRA) subdivided by age group (http://links.lww.com/NEU/D920). **Supplemental Digital Content 3. Supplemental Figure 1.** Total number of **A**, brain MRA/CTA examinations, **B**, CTA examinations, and **C**, MRA examinations in 2005–2019, divided by age group (http://links.lww.com/NEU/D921). **Supplemental Digital Content 4. Supplemental Table 2.** Relative risks (95% Confidence Interval) of brain MRA and CTA examinations adjusted for sex by age group and period (http://links.lww.com/NEU/D922).

## COMMENTS

This paper provides a retrospective review of the detection trends of incidental unruptured aneurysms using noninvasive imaging (CTA and MRA) at a single center in Finland. The authors analyzed 81 261 studies encompassing 48 037 patients from 2005 to 2019. The patients had a mean age of 55.1 years, with 54.2% female and 2.7% having aneurysms. Over time, in addition to the significant increase in the number of scans, the authors found that the detection rate rose from 1.7% (2005-2009) to 3.4% (2015-2019). This increase was primarily noted in patients over 70 years, while the detection rate remained steady for those younger than 50 years. The frequency of detecting small aneurysms (<5 mm) also increased. These findings suggest that the heightened detection of aneurysms is not merely due to broader utilization or enhanced imaging quality but points to a genuine increase in the incidence of aneurysms in the elderly.

The authors correctly identify that it is still being determined how this information should influence clinical practice, including whether treatment should be reserved only for larger aneurysms with a higher risk of rupture. While we typically offer treatment for aneurysms larger than 6 mm, the increase in detection among individuals over 70 years suggests that dynamic changes, such as the emergence of new aneurysms, might carry a heightened risk that would be considered. It is crucial to underscore the increasing prevalence of unruptured intracranial aneurysms in the elderly, emphasizing the urgent need to develop comprehensive guidelines to effectively manage such cases. The fact of the matter is that there is no completely “safe” brain aneurysm: every ruptured aneurysm was unruptured before the actual hemorrhage. With advances in endovascular treatment, which boasts lower morbidity rates than open surgery, there is a growing temptation to consider elective treatment for patients over 70 years. However, it remains unclear whether this strategy effectively alters the natural history of the disease. It is important to note that the aneurysm morphology and location ultimately determine the technique employed for treatment.

**Sheeny V. Vo and Ciarán J. Powers**

*Columbus, Ohio, USA*
