# Supplementary material for: Detection Rates and Trends of Asymptomatic Unruptured Intracranial Aneurysms From 2005 to 2019
Source: Neurosurgery. 2023 Sep 11;94(2):297–306. doi: 10.1227/neu.0000000000002664 (PMC10766300; doi:10.1227/neu.0000000000002664)
Supplement: Supplementary file 1 [file neu-94-297-s001.docx]

**SUPPLEMENTAL DIGITAL CONTENT 1**

**Methods**

**Flow Chart**

All brain CT and MRI examinations identified in 2005-2019 at tertiary hospital

- Patients (n = 130,621)
- Brain CT examinations (n = 182,436)
- Brain MRI examinations (n = 105,900)

**Screening**

Patients excluded

- Without brain MRA/CTA (n = 80,052)
- Brain arteriovenous malformations/fistulas (n = 276)
- Exclusively Fusiform IAs (n = 53)
- Mycotic IAs (n = 2)
- Intracranial pseudoaneurysms (n = 7)
- Non-aneurysmal subarachnoid hemorrhage (n = 124)
- Aneurysmal Subarachnoid Hemorrhage (n = 1479)
- Symptomatic UIAs (n = 27)
- UIAs diagnosed before year 2005 (n = 138)
- UIAs diagnosed outside tertiary hospital (n = 426)

Included examinations in 2005-2019

- Patients (n = 48,037)
- Brain CTA examinations (n = 31,589
- Brain MRA examinations (n = 49,678)
- Patients with UIA (n = 1286)

**Included**

Time period 2010-2014

- Patients with first CTA/MRA (n = 14,190)
- Patients with UIA (n = 368)

Time period 2015-2019

- Patients with first CTA/MRA (n = 20,878)
- Patients with UIA (n = 707)

Time period 2005-2009

- Patients with first CTA/MRA (n = 12,190)
- Patients with UIA (n = 211)

UIA= Unruptured Intracranial Aneurysm

**Identification of Study Population**

Our tertiary hospital utilizes electronic health care information, which is stored in a virtual data warehouse (Auria Clinical Informatics). The virtual data warehouse can produce data tables consisting of all forms of medical care utilization, including radiological imaging, performed in our hospital. We used the Finnish Institute for Health and Welfare coding to search every brain CT and brain MRI examination conducted in our Tertiary Hospital. Each imaging study was labeled with a personalized identification code. The radiological reports of all brain CT and MRI scans were scrutinized to verify if a contrast agent had been administered and intracranial arteries were investigated to ensure that every angiographic examination was included in the study.

**Aneurysm Characteristics**

Unruptured intracranial aneurysms (UIAs) located in the internal carotid artery (ICA) were classified as ophthalmic artery aneurysms, posterior communicating artery aneurysms, anterior choroidal artery aneurysms, and ICA bifurcation aneurysms. Aneurysms in the middle cerebral artery (MCA) were classified as M1 segment aneurysms, M1 bifurcation aneurysms, M1/M2 bifurcation aneurysms, M2 segment aneurysms, and M3-M4 segment aneurysms. Aneurysms in the anterior cerebral artery (ACA) were classified as A1 segment aneurysms, anterior communicating artery aneurysms, and distal ACA aneurysms. Finally, aneurysms in the posterior circulation artery (PCA) were classified as posterior cerebral artery (PCA) P1 segment aneurysms, P2 segment aneurysms, P3 segment aneurysms, basilar tip aneurysms, basilar trunk aneurysms, superior cerebellar artery aneurysms, posterior inferior cerebellar artery aneurysms, and anterior inferior cerebellar artery aneurysms.

We only reported the size of the largest intradural aneurysm or extradural aneurysm if there were no intradural UIAs. UIA sizes were further categorized as 1-2 mm, 3-4 mm, 5-6 mm, 6-9 mm and ≥10 mm.
