# Supplementary material for: Detection Rates and Trends of Asymptomatic Unruptured Intracranial Aneurysms From 2005 to 2019
Source: Neurosurgery. 2023 Sep 11;94(2):297–306. doi: 10.1227/neu.0000000000002664 (PMC10766300; doi:10.1227/neu.0000000000002664)
Supplement: Supplementary file 2 [file neu-94-297-s002.docx]

|  | **Age group, years** | | | | | | | |  |
| --- | --- | --- | --- | --- | --- | --- | --- | --- | --- |
| **Characteristics** | **0-17** | **18-29** | **30-39** | **40-49** | **50-59** | **60-69** | **70-79** | **>79** | **p-value** |
| First CTA/MRA – patients no. | 3053 | 3901 | 4284 | 5368 | 7740 | 9765 | 9051 | 4875 | <.001 |
| Female – no. (%) | 1498 (49.1) | 2464 (63.2) | 2628 (61.3) | 3043 (56.7) | 3950 (51.0) | 4787 (49.0) | 4632 (51.2) | 3024 (62.0) | <.001 |
| Detection rate of UIAs – no. patients (%) |  |  |  |  |  |  |  |  |  |
| *All UIAs* | 11 (0.4) | 24 (0.6) | 55 (1.3) | 139 (2.6) | 251 (3.2) | 341 (3.5) | 301 (3.3) | 164 (3.4) | <.001 |
| *Intradural UIAs* | 5 (0.2) | 16 (0.4) | 48 (1.1) | 120 (2.3) | 237 (3.1) | 329 (3.4) | 289 (3.1) | 156 (2.9) | <.001 |
| *UIAs in female – no. (%)* | 7 (0.5) | 18 (0.7) | 41 (1.6) | 90 (3.0) | 155 (3.9) | 211 (4.4) | 181 (3.9) | 130 (4.3) | <.001^a^ |
| *UIAs in men – no. (%)* | 4 (0.3) | 6 (0.4) | 14 (0.9) | 49 (2.1) | 96 (2.5) | 130 (2.6) | 120 (2.7) | 34 (1.8) |  |
| *Total number of UIAs – no.* | 13 | 29 | 69 | 172 | 315 | 431 | 356 | 180 | <.001 |
| Diameter of the largest aneurysm– mean, mm (SD) | 4.9 (4.8) | 2.6 (1.2) | 4.0 (2.6) | 4.3 (2.5) | 4.5 (3.2) | 4.6 (3.3) | 4.8 (3.2) | 5.3 (4.2) | <.001^b^ |
| Location of largest UIA – no. (%) |  |  |  |  |  |  |  |  |  |
| *ICA* | 0 (0) | 8 (33.3) | 21 (38.2) | 37 (26.6) | 69 (27.5) | 83 (24.3) | 64 (21.3) | 41 (25.0) | .07 |
| *MCA* | 2 (18.2) | 3 (12.5) | 12 (21.8) | 46 (33.1) | 96 (38.2) | 126 (37.0) | 126 (41.9) | 54 (32.9) | .01 |
| *ACA* | 0 (0) | 3 (12.5) | 11 (20.0) | 21 (15.1) | 53 (21.1) | 76 (22.3) | 60 (19.9) | 37 (22.6) | .38 |
| *Posterior circulation* | 2 (18.2) | 2 (8.3) | 4 (7.3) | 14 (10.1) | 14 (5.6) | 33 (9.7) | 32 (10.6) | 17 (10.4) | .48 |
| *Extradural* | 7 (63.6) | 8 (33.3) | 7 (12.7) | 21 (15.1) | 19 (7.6) | 23 (6.7) | 19 (6.3) | 15 (9.1) | <.001 |

**Supplemental Digital Content 2, Table 1.** Patient Characteristics, Detection Rate, and Characteristics of Saccular Unruptured Intracranial Aneurysms (UIAs) from First CT Angiography (CTA) or MR Angiography (MRA) Subdivided by Age Group.

^a^ P-value from generalized linear model adjusted with age groups and time period. Detection rate of UIAs in female compared to male remained significant in every age group (relative risk, 1.66; 95% CI, 1.48-1.89). When adding an interaction with age groups, the p-value was =.11.

^b^ In multivariate logistic regression model adjusted with time period and with separate time period interaction p-value was not significant (p-value=.1).
