# Supplementary material for: Detection Rates and Trends of Asymptomatic Unruptured Intracranial Aneurysms From 2005 to 2019
Source: Neurosurgery. 2023 Sep 11;94(2):297–306. doi: 10.1227/neu.0000000000002664 (PMC10766300; doi:10.1227/neu.0000000000002664)
Supplement: Supplementary file 3 [file neu-94-297-s003.docx]

**Supplemental Digital Content 3, Figure 1.** Total number of brain MRA/CTA examinations (a), CTA examinations (b), and MRA examinations (c) in 2005-2019, divided by age group.
