# Supplementary material for: Detection Rates and Trends of Asymptomatic Unruptured Intracranial Aneurysms From 2005 to 2019
Source: Neurosurgery. 2023 Sep 11;94(2):297–306. doi: 10.1227/neu.0000000000002664 (PMC10766300; doi:10.1227/neu.0000000000002664)
Supplement: Supplementary file 4 [file neu-94-297-s004.docx]

| **Age group** | **2015-2019 vs. 2010-2014** | **2015-2019 vs. 2005-2009** | **2010-2014 vs. 2005-2009** |
| --- | --- | --- | --- |
| MRA examinations |  |  |  |
| *0-17* | **0.77 (0.71-0.82)** | **0.60 (0.55-0.65)** | **0.78 (0.71-0.84)** |
| *18-29* | **0.88 (0.82-0.94)** | **0.86 (0.80-0.93)** | 0.98 (0.90-1.06) |
| *30-39* | **0.94 (0.88-0.99)** | 0.94 (0.87-1.01) | 0.99 (0.92-1.08) |
| *40-49* | **0.92 (0.87-0.97)** | **0.89 (0.83-0.95)** | 0.97 (0.90-1.04) |
| *50-59* | 0.97 (0.92-1.03) | **0.79 (0.75-0.84)** | **0.82 (0.76-0.87)** |
| *60-69* | 0.99 (0.94-1.04) | **1.23 (1.16-1.31)** | **1.24 (1.16-1.33)** |
| *70-79* | **1.43 (1.35-1.52)** | **1.73 (1.60-1.86)** | **1.21 (1.11-1.31)** |
| *>79* | **1.42 (1.27-1.58)** | **2.38 (2.03-2.79)** | **1.68 (1.41-1.99 )** |
| CTA examinations |  |  |  |
| *0-17* | **0.69 (0.48-0.97)** | **0.46 (0.33-0.65)** | **0.67 (0.47-0.97)** |
| *18-29* | **0.72 (0.61-0.85)** | **0.47 (0.40-0.55)** | **0.66 (0.55-0.78)** |
| *30-39* | **0.80 (0.70-0.91)** | **0.66 (0.58-0.76)** | **0.83 (0.72-0.96)** |
| *40-49* | 0.93 (0.85-1.03) | **0.77 (0.70-0.85)** | **0.83 (0.74-0.93)** |
| *50-59* | 0.97 (0.90-1.05) | 0.69 (0.64-0.75) | 0.71 (0.65-0.78) |
| *60-69* | 0.84 (0.80-0.90) | **1.08 (1.01-1.16)** | **1.28 (1.19-1.38)** |
| *70-79* | **1.19 (1.12-1.26)** | **1.28 (1.19-1.36)** | 1.07 (0.99-1.16) |
| *>79* | **1.19 (1.11-1.27)** | **1.49 (1.38-1.62)** | **1.26 (1.15-1.38)** |

**Supplemental Digital Content 4, Table 2.** Relative Risk (95% Confidence Interval) of Brain MRA and CTA Examinations Adjusted for Sex by Age Group and Time Period.

Numbers in bold indicate statistically significant results (p<.05).
